# Prioritising positively selected variants in whole-genome sequencing data using *FineMAV*

**DOI:** 10.1186/s12859-021-04506-9

**Published:** 2021-12-18

**Authors:** Fadilla Wahyudi, Farhang Aghakhanian, Sadequr Rahman, Yik-Ying Teo, Michał Szpak, Jasbir Dhaliwal, Qasim Ayub

**Affiliations:** 1 School of Science, Monash University Malaysia, 47500 Bandar Sunway, Selangor Darul Ehsan Malaysia; 2 Monash University Malaysia Genomics Facility, 47500 Bandar Sunway, Selangor Darul Ehsan Malaysia; 3 Present Address: Genes and Human Disease Research Program, Oklahoma Medical Research Foundation,, Oklahoma City, OK 73104 USA; 4 Tropical Medicine and Biology Multidisciplinary Platform, Monash University Malaysia, 47500 Bandar Sunway, Selangor Darul Ehsan Malaysia; 5 Saw Swee Hock School of Public Health, National University of Singapore, Singapore, Singapore; 6 European Bioinformatics Institute, Hinxton, CB10 1SA UK; 7 Wellcome Sanger Institute, Wellcome Genome Campus, Hinxton, CB10 1SA UK; 8 School of Information Technology, Monash University Malaysia, 47500 Bandar Sunway, Selangor Darul Ehsan Malaysia

**Keywords:** Adaption, Evolutionary genomics, Human evolution, Population differentiation, Selective sweep

## Abstract

**Background:**

In population genomics, polymorphisms that are highly differentiated between geographically separated populations are often suggestive of Darwinian positive selection. Genomic scans have highlighted several such regions in African and non-African populations, but only a handful of these have functional data that clearly associates candidate variations driving the selection process. Fine-Mapping of Adaptive Variation *(FineMAV)* was developed to address this in a high-throughput manner using population based whole-genome sequences generated by the 1000 Genomes Project. It pinpoints positively selected genetic variants in sequencing data by prioritizing high frequency, population-specific and functional derived alleles.

**Results:**

We developed a stand-alone software that implements the *FineMAV* statistic. To graphically visualise the *FineMAV* scores, it outputs the statistics as bigWig files, which is a common file format supported by many genome browsers. It is available as a command-line and graphical user interface. The software was tested by replicating the *FineMAV* scores obtained using 1000 Genomes Project African, European, East and South Asian populations and subsequently applied to whole-genome sequencing datasets from Singapore and China to highlight population specific variants that can be subsequently modelled. The software tool is publicly available at https://github.com/fadilla-wahyudi/finemav.

**Conclusions:**

The software tool described here determines genome-wide *FineMAV* scores, using low or high-coverage whole-genome sequencing datasets, that can be used to prioritize a list of population specific, highly differentiated candidate variants for in vitro or in vivo functional screens. The tool displays these scores on the human genome browsers for easy visualisation, annotation and comparison between different genomic regions in worldwide human populations.

**Supplementary Information:**

The online version contains supplementary material available at 10.1186/s12859-021-04506-9.

## Background

Human whole-genome sequencing projects have contributed to the advancement of population genomics, specifically the unbiased detection of positive selection in human populations. In comparison to genotyping, sequencing mitigates ascertainment bias and captures greater genomic variation [1] making it suitable for selection scans. The rise in population-based sequencing initiatives has garnered an interest in the study of positive selection, because identifying genetic variants that are positively selected can provide insight into new molecular functions that come with adaptation. These selective scans have provided vast lists of genes and variants and except for a few classical examples [2], it has been difficult to identify potentially functional variants that should be followed up in vitro or in vivo models.

Fine-Mapping of Adaptation Variation (*FineMAV*) is a statistical method that prioritizes functional SNP candidates under selection and depends upon population differentiation [3]. It pinpoints candidate positively selected variants at putative loci in a high-throughput manner, thus, enabling the modelling of such variants in vitro or in vivo [3]. *FineMAV* was developed to overcome a challenge that existing positive selection statistical methods face in that they are unable to distinguish between neutral, hitchhiked variants and true positively selected variants [3]. *FineMAV* does this by incorporating methods that detect regions showing signatures of positive selection (population differentiation and high frequency of derived alleles) and subject these regions to functional annotation under the assumption that it is unlikely for a deleterious or functional variant to reach high frequency in a given randomly mating population unless it confers some sort of functional advantage [3].

To measure population differentiation, *FineMAV* employs a derived allele purity (*DAP*) equation to describe the disparate spread of derived alleles across populations [3]. The derived allele frequency (*DAF*) equation is used to determine sites with high frequency of derived alleles [3]. To annotate functionality, *FineMAV* uses the Combined Annotation-Dependent Depletion (CADD) method which takes into account multiple variant annotations and condenses it into a single score called the C score [4]. The C scores predict whether a SNP or indel in the human genome is functional, deleterious or pathogenic [4]. The phred-scaled C scores (CADD_PHRED) are expressed as rankings relative to all possible substitutions of the human genome and range from 1 to 99 [4]. For example, a variant that scores more than 10 would be within the top 10% of potentially deleterious substitutions. A score of 20 would indicate the top 1% and 30 would be 0.1% and so on [4]. Incorporating CADD scores can, therefore, differentiate between neutral alleles, which are predicted as non-deleterious, and true positively selected alleles, which are predicted as effectively functional or deleterious [3]. In this article, we introduce a stand-alone application that can perform *FineMAV* calculations on whole-genome sequencing data and can output bigWig files which can be used to graphically visualise the scores on genome browsers. We test the software using the 1000 Genomes Project phase 3 dataset [5] and whole-genome sequencing datasets from Singapore and China [6–8].

## Implementation

### Pipeline

**Fig. 1:**
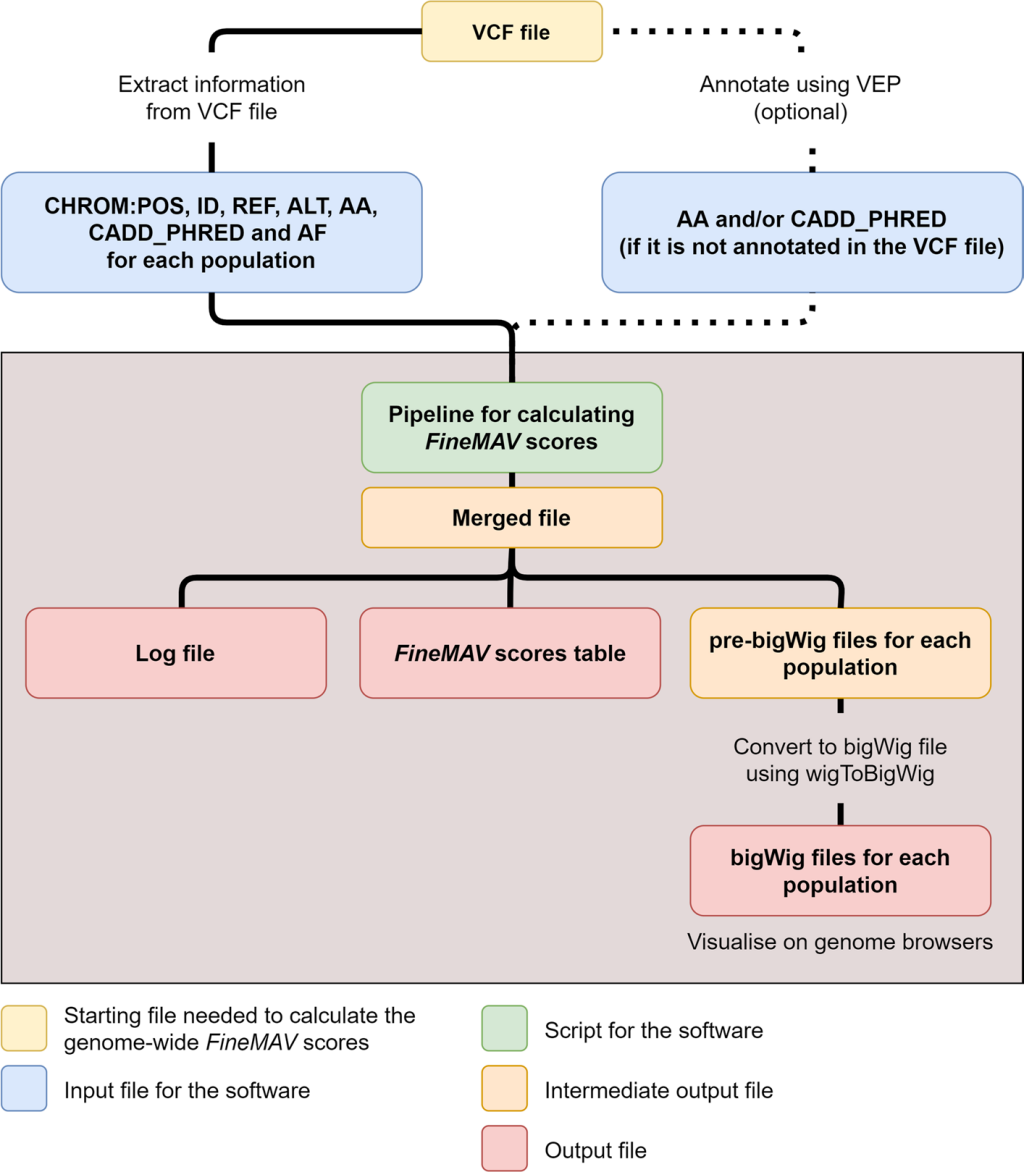
Pipeline for calculating the genome-wide *FineMAV* scores. The boxed region highlighted in grey are the parts of the workflow that are automated by the software. The intermediate output files are deleted when the pipeline is complete. AA: ancestral allele, CADD_PHRED: phred-scaled Combined Annotation Dependent Depletion scores, VEP: Variant Effect Predictor, AF: allele frequency of the alternative allele

The Python-based *FineMAV* software works with high-throughput, massively parallel, sequencing data and relies on the information that can be extracted from variant call format (VCF) files (version 4.2 and above) (Table 1). The Python script can be found in Additional file 1. The pipeline for the software is illustrated in Fig. 1. We recommend users to use jointly-called, multi-sample genomic VCF (gVCF) as it reports every site in the genome regardless of whether they carry variation or not. This is preferable for *FineMAV* analysis as it can distinguish between sites that are homozygous for the reference allele and those with missing data.

**Table 1:** Information needed for the tab-delimited input files

Information needed from the VCF file	Description	Mandatory VCF column
CHROM:POS	Chromosome number:position	Yes
ID	Identifier	Yes
REF	Reference base	Yes
ALT	Alternative base	Yes
AA	Ancestral allele	No
CADD_PHRED	Phred-scaled Combined Annotation Dependent Depletion (CADD) score	No
AF	Allele frequency (AF) for the alternative base. The AF should be reported for each population	No

This information can be extracted from the VCF file and provided in a tab-delimited format for the software to calculate the *FineMAV* scores

### Input files and dataset

The *FineMAV* software requires the user to provide the input data from the VCF file in a tab-delimited file format (Table 1). We recommend extracting the information utilising the BCFtools [9] query command. Some of the information mentioned in Table 1 can be found in the INFO column and are not mandatory in VCF files.

In instances where the allele frequency (AF) for each population is not annotated in the INFO column, we suggest using the BCFtools fill-tags plugin to determine the AF first, and then piping it to the BCFtools query command for extraction. If the ancestral allele and/or the CADD_PHRED are not available in the VCF file, the software allows the user to supplement this information using the Ensembl Variant Effect Predictor (VEP) [10]. The output file must be tab-delimited and the first column must be the “Location”, which indicates the position of the variant using the standard coordinate format (i.e. chromosome:start).

We tested our implementation code by generating genome-wide *FineMAV* scores using the 1000 Genomes Project low coverage whole-genome sequences from African, European, East and South Asian continental populations to replicate the published results [3]. Subsequently, we used the pipeline to calculate *FineMAV* scores for high-coverage whole-genome sequencing datasets generated by the Singapore Sequencing Indian Project (SSIP) [6], the Singapore Sequencing Malay Project (SSMP) [7] and a dataset of 90 Han Chinese individuals (90HC), that included 83 samples from the 1000 Genomes Project [8].

### Calculating the genome-wide *FineMAV* score

The *FineMAV* score was estimated for genome-wide single nucleotide polymorphisms (SNPs) as only the ancestral allele could be unambiguously determined only for this class of variations. The derived allele for each SNP was calculated by multiplying three metrics: *DAP*, *DAF* and the CADD_PHRED score [3]. *DAP* was calculated for each variant using the following equations: ${d}_{N}= \sum_{i=1}^{n}{d}_{i}$, ${f}_{i}= \frac{{d}_{i}}{{d}_{N}}$ and $DAP= \sum_{i=1}^{n}{f}_{i}^{x}$ respectively, where $n$ is the number of populations, ${d}_{i}$ is the derived allele count in one population $i$ where $i\in \{1, 2\dots , n\}$ and $x$ is the penalty parameter used to penalize allele sharing between the populations [3]. The aforementioned *DAP* equation is used when the population sizes are equal. In this software, the *DAP* is calculated using the derived allele frequency instead of counts in order to take into account population sizes that might be different.

The penalty parameter $x$ was determined empirically by Szpak et al. [3] for varying number of $n$ populations, ranging from 2 to 7 (Table 2). However, should the user intend to analyse more than 7 populations or decide on another value for $x$, they are able to change it.

**Table 2:** Recommended minimal value of the penalty parameter ($x$), rounded off to two decimal places, for a given $n$ as determined by Szpak et al. [3]

Number of populations ($n$)	Penalty parameter ($x$)
2	4.96
3	3.50
4	2.98
5	2.71
6	2.53
7	2.41

**Fig. 2:**
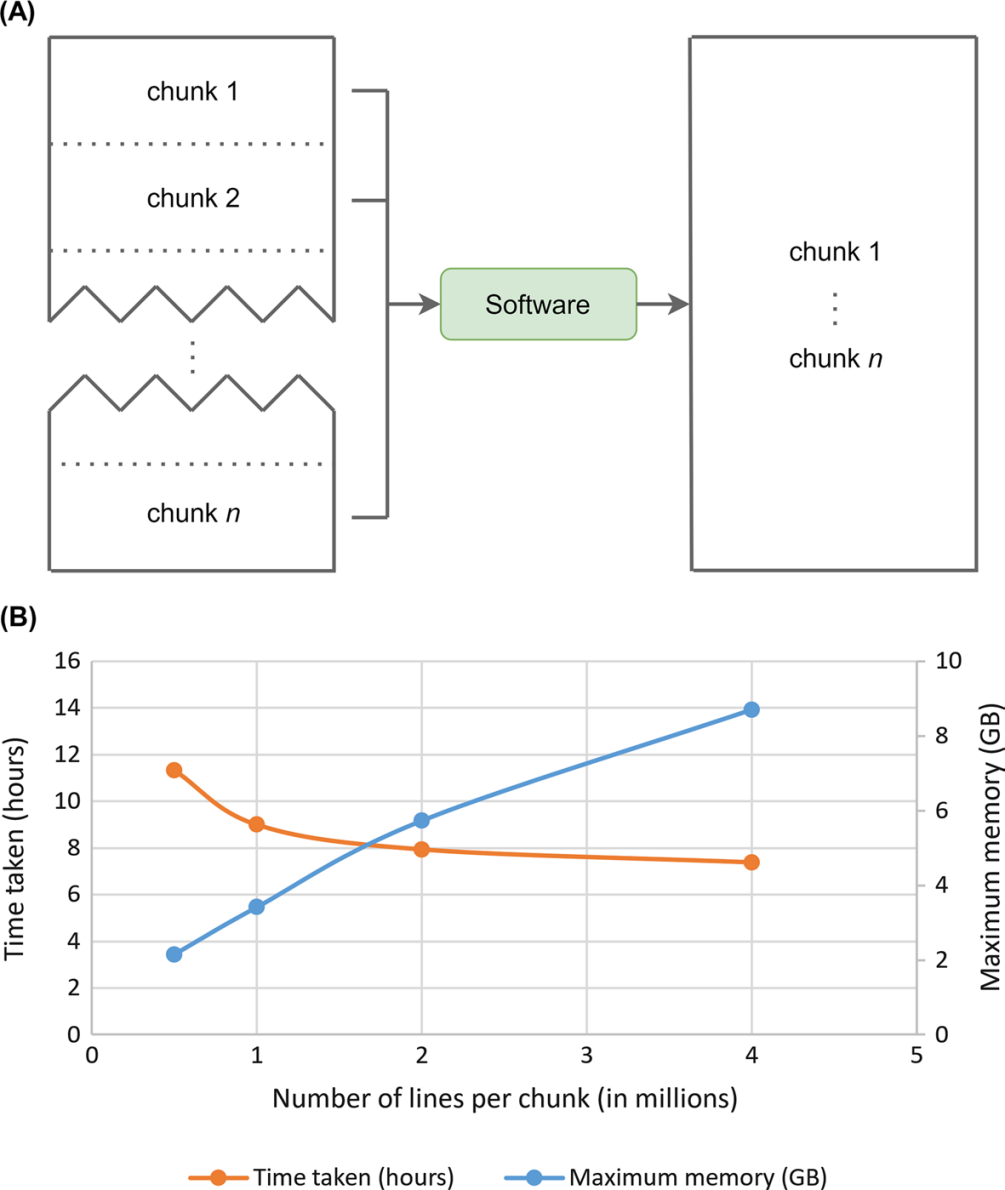
Utilising the chunk size option. **A** Diagram illustrating how the software separates the input files into chunks and iterates through them when performing the *FineMAV* calculations. It proceeds to merge them into one output file. **B** Utilising the chunk size option. A graph that compares the time taken and the maximum random access memory (RAM) when different chunk sizes for a dataset of 66,236,516 biallelic SNPs is used

*FineMAV* calculations are done by splitting the file(s) into smaller chunks to optimise the random access memory (RAM) usage (Fig. 2A). The default size of the chunk is 200,000 lines. However, the user can specify the chunk size they require. We also tested the performance of the chunk size option using 66,236,516 biallelic SNPs across four population groups. Computational experiments were run on Ubuntu 16.04 LTS with a 3.60 GHz 8-core Intel Core i7-4790 processor with 31.3 GB RAM and 950.6 GB of hard disk memory. The size of the input file, which contains the data extracted from the VCF file, and the VEP-generated file was 2.0 GB and 2.1 GB respectively. Figure 2B illustrates the maximum RAM usage and the time taken when different chunk sizes are utilised. As expected, the larger the chunk size, the faster the run time, up to a certain point. The optimal chunk size would vary depending on the size of the input files and the computing power.

### Generating the output files

The software produces three kinds of output files: a log file, a tab-delimited file containing the genome-wide *FineMAV* scores along with the intermediate calculations and a bigWig file. The bigWig [11] is a common file format used for graphical visualisation on downloadable and online genome browsers such as Ensembl or the UCSC genome browsers [12, 13].

## Results and discussion

We initially tested our implementation code by generating genome-wide *FineMAV* scores using the 1000 Genomes Project African, European, East and South Asian continental populations [5] and obtained *FineMAV* scores that were significantly correlated with the published data (Spearman’s correlations ≥ 0.9999 and the *p*-values < 0.00001) for all four continental populations [3]. When comparing the top 100 *FineMAV* outliers across all four continental populations with the published data, only 5/300 variants did not overlap with the published results and all five of these variants were missing, because they did not pass our data filtering criteria.

**Fig. 3:**
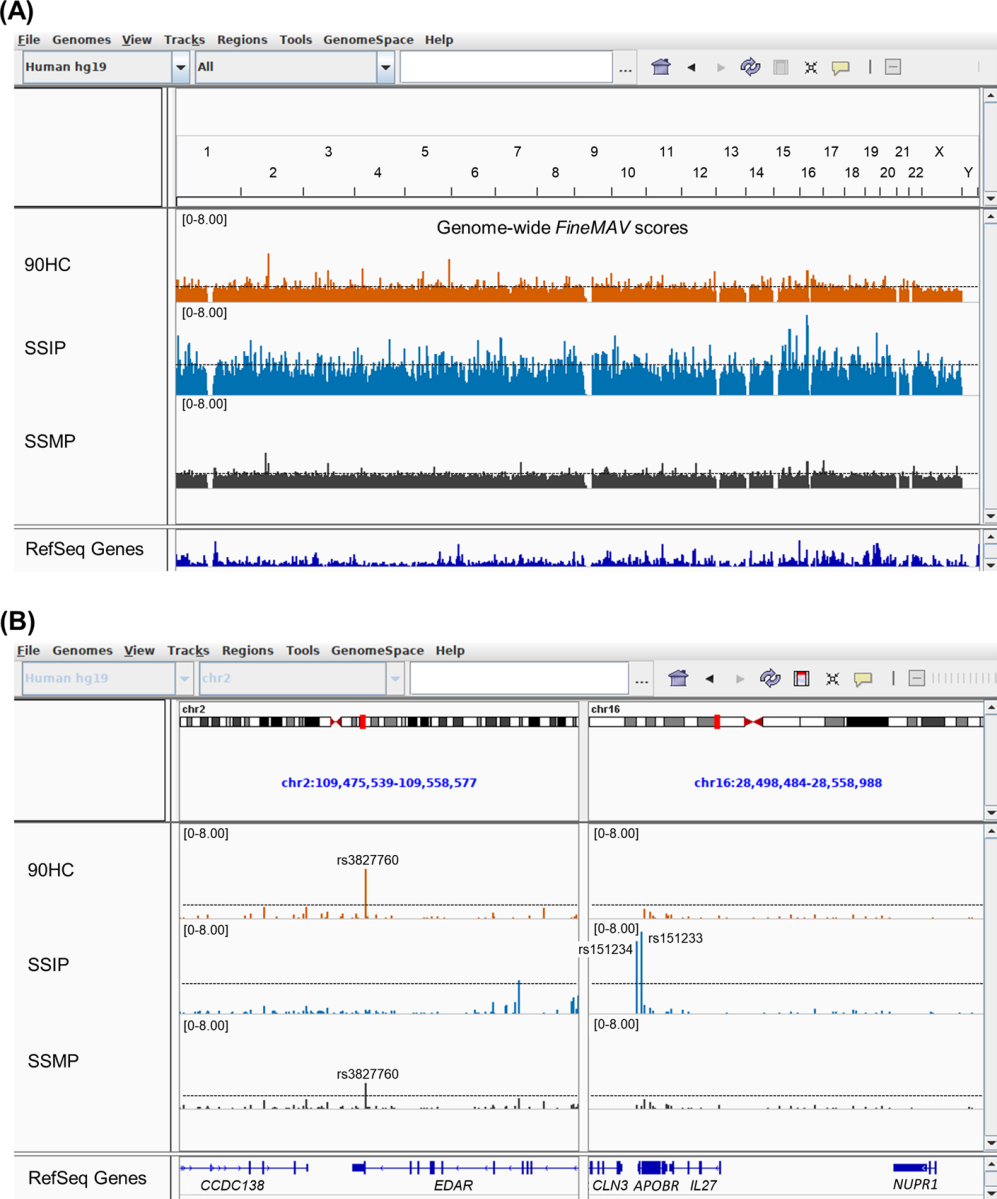
Annotated screenshot of the bigWig files of the genome-wide *FineMAV* scores. **A** *FineMAV* scores for Han Chinese (90HC, orange), Singaporean Indian (SSIP, blue) and Singaporean Malay (SSMP, grey) populations displayed on the Integrative Genomics Viewer (IGV). The genomic regions on display are the autosomal and the X chromosomes and the horizontal line depicts the 99th percentile. **B** A multi-locus view of two regions where the left panel displays a locus with a well-known positively selected missense variant in *EDAR* (rs3827760) in East Asians that also stands out in the SSMP population. The right panel displays a novel locus with two high scoring variants in SSIP: rs151233, a synonymous variant in *APOBR* and rs151234, an intronic variant in *CLN3* that stand out in the SSIP

Next, we used the pipeline to calculate *FineMAV* scores for three high-coverage whole-genome sequencing datasets: the SSIP [6], SSMP [7] and 90HC [8]. These datasets were mapped to the GRCh37/hg19 reference genome. The VCF files for the autosomal and the X chromosome were merged and filtered to select high-quality biallelic sites that were variable in all three populations. This resulted in a final VCF file containing 5,748,704 SNPs in which the CHROM:POS, ID, REF, ALT and AF for each population were retrieved and stored in a tab-delimited file. As the VCF files did not contain the CADD_PHRED nor the ancestral alleles, a separate tab-delimited file containing this information for these SNPs was generated by leveraging the Ensembl VEP’s plugins. For CADD_PHRED annotation, we used the CADD version (v1.4) for the reference genome GRCh37/hg19 (https://krishna.gs.washington.edu/download/CADD/v1.4/GRCh37/whole_genome_SNVs.tsv.gz) [4, 14] The FASTA files containing the ancestral sequences were downloaded from the Ensembl webpage using the following URL: ftp://ftp.ensembl.org/pub/release75/fasta/ancestral_alleles/homo_sapiens_ancestor_GRCh37_e71.tar.bz2 [15, 16]. These two files were then fed to the software to produce bigWig files which can be visualised in genome browsers as presented in Fig. 3.

The *FineMAV* statistic was able to replicate known positively selected variants as well as pinpoint novel ones from the three populations (Table 3, Additional file 2). Examples of known variants included the derived alleles for rs3827760 (NC_000002.12:g.108897145A > G, ENSP00000258443.2:p.Val370Gly) in ectodysplasin A receptor (*EDAR*) and rs2293766 (NC_000007.13:g.100371358G > A, ENSP00000423579.1:p.Trp1883Ter) in zonadhesin (*ZAN*). These variants have also been highlighted previously in several genomic scans for selection in East Asian populations [3, 17, 18] and in this study, they were high scoring in the 90HC and SSMP (Table 3). Several studies that have looked at the missense variant rs3827760 in *EDAR* have confirmed its pleiotropic effects. The non-synonymous mutation has been found to be associated with hair thickness [19–21], shovel-shaped incisors [22–24], ear morphology [25, 26], increased density of eccrine sweat glands, reduced mammary fat pad and increased mammary ductal gland branching [21] and despite extensive research, it still remains unclear as to why this allele is positively selected in the region. Some have theorised that increased sweat gland density resulted in better thermoregulation during warmer climates or that perhaps male sexual preference may have played a role in its selection [21]. Others hypothesise that selection for increased mammary gland branching would lead to better mother-to-child nutrient transfer, especially for vitamin D, to prevent vitamin D deficiency in regions with lower ultraviolet (UV) levels [27]. *ZAN* encodes an acrosomal protein in the sperm called zonadhesin. A study employing *Zan* knockout mice found that their sperms remained fertile and had increased adhesion to the jelly-like coating of the egg (zona pellucida) of other species like pig, cow and rabbit [28]. As *ZAN* is responsible for species-specific binding, it can be speculated that a truncation, as a result of the nonsense mutation in rs2293766, could have mediated interbreeding between archaic humans and modern humans in Asia [29].

**Table 3:** Top 10 *FineMAV* candidates from the Han Chinese (90HC), Singaporean Indian (SSIP) and Singaporean Malay (SSMP) populations

Chr	Position ^a^	SNP ID	Gene	Consequence ^b^	*DAF* 90HC	*DAF* SSIP	*DAF* SSMP	*FineMAV*	Known or novel
*90HC*									
2	109513601	rs3827760:A > G	*EDAR*	Missense (p.Val370Ala)	0.922	0.029	0.490	4.661	Known [3, 17, 18]
5	176099727	rs13186794:A > G	*–*	Intergenic	0.494	0.057	0.047	4.114	Novel
5	176099728	rs13186795:A > G	*–*	Intergenic	0.494	0.057	0.057	4.096	Novel
4	31442427	rs56345433:G > A	*–*	Intergenic	0.528	0.086	0.021	3.211	Novel
3	98031307	rs2316271:T > A	*OR5H8*	Stop gained (p.Leu184Ter)	0.767	0.314	0.599	3.102	Novel
16	31088347	rs749671:G > A	*ZNF646*	Synonymous (p.Glu234 =)	0.906	0.043	0.776	3.053	Known [18]
5	76129053	rs631465:T > C	*F2RL1*	Synonymous (p.Ile207 =)	0.522	0.014	0.208	3.008	Novel
2	109451118	rs72627476:A > G	*CCDC138*	Intronic	0.917	0.029	0.484	2.961	Known [3]
12	132106717	rs10794470:T > C	*AC117500.3*	Intronic	0.272	0.000	0.005	2.940	Novel
7	14587199	rs10236893:G > A	*DGKB*	Intronic	0.417	0.029	0.120	2.895	Novel
*SSIP*									
16	28506428	rs151233:C > T	*APOBR*	Synonymous (p.Leu22 =)	0.006	0.571	0.026	7.677	Novel
16	30936081	rs35675346:G > A	*FBXL19*	Missense (p.Glu10Lys)	0.061	0.800	0.188	7.213	Known [18, 43]
16	28505660	rs151234:G > C	*CLN3*	Intronic	0.006	0.571	0.031	6.839	Novel
16	31044683	rs58726213:A > G	*STX4*	Upstream gene	0.089	0.871	0.214	6.686	Known [18, 43]
15	64592833	rs114713921:T > C	*CSNK1G1*	5 prime UTR	0.006	0.486	0.036	6.341	Novel
16	30666367	rs3747481:C > T	*PRR14*	Missense (p.Pro359Leu)	0.100	0.857	0.245	6.090	Known [18]
19	49206674	rs601338:G > A	*FUT2*	Stop gained (p.Trp154Ter)	0.011	0.186	0.016	6.033	Known [44]
15	91452595	rs2106673:A > G	*MAN2A2*	Missense (p.Gln412Arg)	0.017	0.514	0.063	5.746	Novel
10	17407147	rs729170:G > T	*ST8SIA6*	Intronic	0.006	0.343	0.005	5.736	Novel
15	64653984	rs8026043:G > T	*PCLAF*	Downstream gene	0.006	0.486	0.036	5.726	Novel
*SSMP*									
2	98272491	rs2290123:A > G	*ACTR1B*	3 prime UTR	0.033	0.029	0.380	3.378	Known [18]
2	97613974	rs114979404:C > G	*FAM178B*	Intronic	0.022	0.029	0.375	2.806	Known [18]
17	2238152	rs79597880:T > C	*TSR1*	Missense (p.Lys199Glu)	0.089	0.014	0.297	2.747	Novel
16	31088347	rs749671:G > A	*ZNF646*	Synonymous (p.Glu234 =)	0.906	0.043	0.776	2.616	Known [18]
7	100371358	rs2293766:G > A	*ZAN*	Stop gained (p.Trp1883Ter)	0.528	0.257	0.557	2.531	Known [3, 45]
2	109513601	rs3827760:A > G	*EDAR*	Missense (p.Val370Ala)	0.922	0.029	0.490	2.474	Known [3, 17, 18]
3	98031307	rs2316271:T > A	*OR5H8*	Stop gained (p.Leu184Ter)	0.767	0.314	0.599	2.424	Novel
11	62848487	rs11231341:A > C	*SLC22A24*	Stop gained (p.Tyr501Ter)	0.867	0.757	0.792	2.421	Novel
12	57865558	rs2229300:G > T	*GLI1*	Missense (p.Gly1012Val)	0.050	0.014	0.224	2.402	Novel
16	31075175	rs2303223:G > A	*ZNF668*	Synonymous (p.Gly225 =)	0.911	0.043	0.781	2.290	Known [3]

^a^The genomic position according to the GRCh37/hg19 reference genome

^b^The most severe variant consequence according to Ensembl

Chr: chromosome, *DAF*: derived allele frequency, UTR: untranslated region

As seen in Fig. 3, the Singaporean Indian population have more population-specific signals than the Han Chinese and Singaporean Malay populations. This is because the Han Chinese and Singaporean Malays are genetically more closely related to each other than the Singaporean Indian population [18], and *FineMAV* penalizes allele sharing between populations and highlights high frequency population-specific mutations. Some of the highest-scoring SNPs observed in SSIP are located in chromosome 16 (Fig. 3, Table 3). We suspected that this could be due to the effect of genetic hitchhiking, driven by the selection of rs201075024 (NC_000016.9:g.31099000C > T, ENSP00000280606.6:p.Gly34Ser) (*PRSS53*), a SNP that has been reported to be positively selected in South Asian populations and was missing in our dataset [3]. *PRSS53* encodes a serine protease and is expressed in hair follicles [30] and rs201075024 lies 10 base pairs away from rs11150606 (NC_000016.9:g.31099011 T > C, ENSP00000280606.6:p.Gln30Arg), another SNP in the same gene that is positively selected in East Asians [3, 30] and has been associated with hair shape in Latin Americans [30]. The functional effects of the rs201075024 missense mutation on the serine protease is still unknown. However, based on previous publications, it can be hypothesised that this variant also influences hair shape in South Asians. rs201075024 (*PRSS53*) was excluded from this selection scan because it was not polymorphic in all three datasets. Within the top 10 *FineMAV* candidates in SSIP, three SNPs are in linkage disequilibrium with rs201075024 (*PRSS53*): rs35675346 (NC_000016.9:g.30936081G > A, ENSP00000369666.2:p.Glu10Lys) (*FBXL19*), rs58726213 (NC_000016.9:g.31044683A > G) (*STX4*) and rs3747481 (NC_000016.9:g.30666367C > T, ENSP00000300835.4:p.Pro359Leu) (*PRR14*), with r^2^ values of 0.45, 0.26 and 0.16, respectively. This suggests that the three SNPs may be neutral and tagging the *PRSS53* rs201075024 variant. Interestingly, according to the Genotype-Tissue Expression (GTEx) database (V8 release), these SNPs were reported as expression and splicing quantitative trait loci (eQTL/sQTL) for *PRSS53* in various tissues [31].

Examples of novel SNPs that were identified in this study included two missense mutations: rs79597880 (NC_000017.10:g.2238152 T > C, ENSP00000301364.4:p.Lys199Glu) in the pre-rRNA-processing protein TSR1 homolog (*TSR1*) and rs2229300 (NC_000012.11:g.57865558G > T, ENSP00000228682.2:p.Gly1012Val) in glioma-associated oncogene family zinc finger 1 (*GLI1*) in the Singaporean Malay population (Table 3). So far, the effects of these SNPs on their respective proteins are unknown. The exact function of *TSR1* is yet to be elucidated, but it plays a role in ribosome maturation [32]. It was reported that several rare (minor allele frequency < 1%) mutations of this gene, including missense mutations, may be associated with spontaneous coronary artery dissection (SCAD), a condition where the coronary artery tears resulting in two lumens: the true lumen and a false one [33]. However, there are no functional studies to confirm this association. *GLI1*, on the other hand, is a well-established oncogene and its protein is a drug target for several anti-cancer medications [34]. According to the Catalogue of Somatic Mutations in Cancer (COSMIC), 65.55% of mutations that are observed in *GLI1* are missense substitutions [35]. The missense mutation rs2229300 is listed as an entry in COSMIC (COSV57366104) and was found in 14 tissue samples: 12 in the prostate [36], one in the large intestine and one in the lung [35].

It should be noted that admixture, or shared ancestry, can result in less population-specific signals [3]. This could mean that non-population-specific variants can be ranked high if the variants are highly deleterious and, therefore, have a high CADD_PHRED score. This is true for the derived allele in rs11231341 (NC_000011.9:g.62848487A > C, ENSP00000396586.1:p.Tyr501Ter) (*SLC22A24*) in which the global allele frequency is 0.75 [5] but was the 8^th^ highest scoring allele in Singaporean Malays (Table 3), a highly admixed population.

## Conclusions

**Fig. 4:**
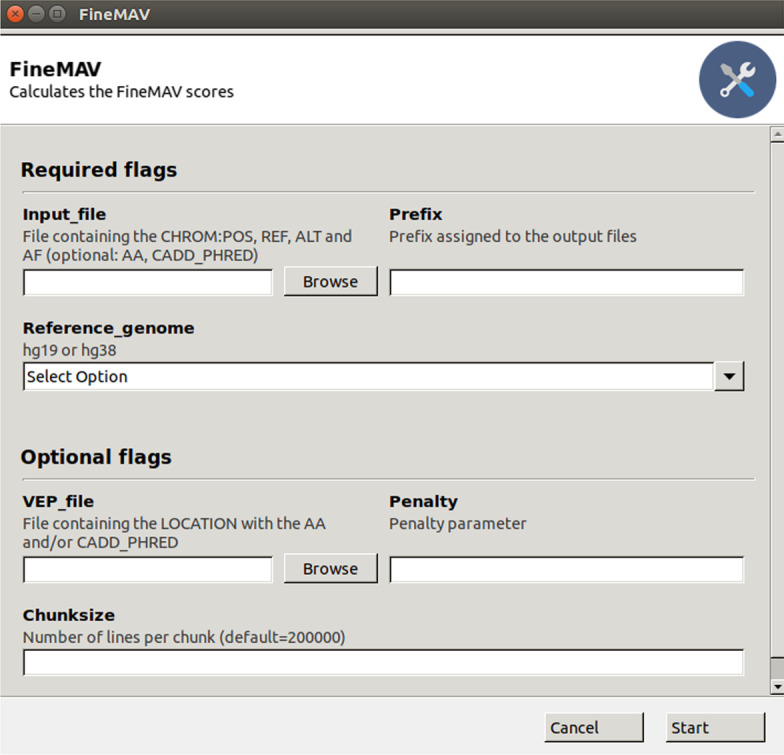
Screenshot of the *FineMAV* software as a graphical user interface

We developed a user-friendly command line and graphical user interface (Fig. 4) platform to enable determination of genome-wide *FineMAV* scores using whole-genome sequencing datasets and to subsequently display these scores on genome browsers. This allows for easy, visual comparison between different genomic regions and human populations. It is designed to leverage on familiar bioinformatics tools and genome browsers, to be memory-efficient in anticipation of larger worldwide population sequencing datasets [37–42].

## Availability and requirements

Project name: *FineMAV*.

Project home page: https://github.com/fadilla-wahyudi/finemav.

Operating systems: Linux.

Programming language: Python.

Other requirements: None.

License: MIT License.

Any restrictions to use by non-academics: None.

## Supplementary Information

**Additional file 1.** Source code for the *FineMAV* program.

**Additional file 2.** Table containing the top 50 *FineMAV* candidates from the Han Chinese (90HC), Singaporean Indian (SSIP) and Singaporean Malay (SSMP) populations.

## Data Availability

The software tool is publicly available on GitHub (https://github.com/fadilla-wahyudi/finemav). The datasets analysed during the current study are available from the following links: 1000 Genomes Project Phase 3 dataset (ftp://ftp.1000genomes.ebi.ac.uk/vol1/ftp/release/20130502/), Sequencing of 90 Han Chinese genomes (90HC) (https://www.ebi.ac.uk/ena/data/view/PRJEB20820), Singapore Sequencing Indian Project (SSIP) (https://blog.nus.edu.sg/sshsphphg/singapore-sequencing-indian/), and Singapore Sequencing Malay Project (SSMP) (https://blog.nus.edu.sg/sshsphphg/singapore-sequencing-malay/).

## Abbreviations

90HC: Whole-genome sequencing dataset of 90 Han Chinese individuals
AF: Allele frequency
CADD: Combined Annotation-Dependent Depletion
CADD_PHRED: Phred-scaled C score
*DAF*: Derived allele frequency
*DAP*: Derived allele purity
*FineMAV*: Fine-Mapping of Adaptive Variation
gVCF: Genome variant call format
RAM: Random access memory
SNP: Single nucleotide polymorphism
SSIP: Singapore Sequencing Indian Project
SSMP: Singapore Sequencing Malay Project
VCF: Variant call format
VEP: Variant Effect Predictor
